# Insecticide Control of Vector-Borne Diseases: When Is Insecticide Resistance a Problem?

**DOI:** 10.1371/journal.ppat.1001000

**Published:** 2010-08-05

**Authors:** Ana Rivero, Julien Vézilier, Mylène Weill, Andrew F. Read, Sylvain Gandon

**Affiliations:** 1 Génétique et Evolution des Maladies Infectieuses (UMR CNRS 2724), Centre de Recherche IRD, Montpellier, France; 2 Institute des Sciences de l'Evolution de Montpellier (UMR CNRS 5554), Université de Montpellier II, Montpellier, France; 3 Center for Infectious Disease Dynamics, Departments of Biology and Entomology, Pennsylvania State University, University Park, Pennsylvania, United States of America; 4 Fogarty International Center, National Institutes of Health, Bethesda, Maryland, United States of America; 5 Centre d'Ecologie Fonctionnelle et Evolutive (UMR CNRS 5175), Montpellier, France; University of California San Diego, United States of America

## Abstract

Many of the most dangerous human diseases are transmitted by insect vectors. After decades of repeated insecticide use, all of these vector species have demonstrated the capacity to evolve resistance to insecticides. Insecticide resistance is generally considered to undermine control of vector-transmitted diseases because it increases the number of vectors that survive the insecticide treatment. Disease control failure, however, need not follow from vector control failure. Here, we review evidence that insecticide resistance may have an impact on the quality of vectors and, specifically, on three key determinants of parasite transmission: vector longevity, competence, and behaviour. We argue that, in some instances, insecticide resistance is likely to result in a decrease in vector longevity, a decrease in infectiousness, or in a change in behaviour, all of which will reduce the vectorial capacity of the insect. If this effect is sufficiently large, the impact of insecticide resistance on disease management may not be as detrimental as previously thought. In other instances, however, insecticide resistance may have the opposite effect, increasing the insect's vectorial capacity, which may lead to a dramatic increase in the transmission of the disease and even to a higher prevalence than in the absence of insecticides. Either way—and there may be no simple generality—the consequence of the evolution of insecticide resistance for disease ecology deserves additional attention.

## Introduction

Vector-borne diseases are among the major causes of illness and death, particularly in tropical and subtropical countries. Vector control, through the use of insecticides, plays a key role in the prevention and control of infectious diseases such as malaria, dengue, and filariasis [1]. The widespread use of insecticides can, however, lead to the development of insecticide resistance, making insecticide use ineffective and limiting the available options for disease control [2]. Insecticide resistance, including resistance to multiple types of insecticides, has arisen in all the insect species that are the major vectors of human diseases (Table 1). Consequently, insecticide resistance is considered a serious public health challenge.

**Table 1:** Insecticide resistance mechanisms reported to date in natural populations of the main insect vectors of human diseases.

Vector	Pathogen (Disease)	Insecticide Resistance	
		Metabolic	Target Site
**Diptera (mosquitoes, flies)**			
*Aedes* sp.	*Brugia, Wuchereria* (lymphatic filariasis), yellow fever virus, dengue virus, encephalitis virus	EST [37] GST [37]	SCH [37] GABA[37]
*Anopheles* sp.	*Plasmodium* sp. (malaria), *Wuchereria* (filariasis)	EST [37] GST [37], [118] MOX [37]	SCH [37], [118] AChE [37], [118] GABA [114]
*Culex* sp.	*Wuchereria* (filariasis), West Nile virus, encephalitis virus	EST [37] GST [114] MOX [37]	SCH [114] AChE [37] GABA [114]
*Phlebotomus* sp.	*Leishmania* sp. (leishmaniasis)	EST [119]	AChE [119]
*Simulium* sp.	*Onchocerca* sp. (river blindness)	EST [120], [121]	-
**Haemiptera (true bugs)**			
*Rhodnius* sp.	*Trypanosoma* sp. (Chagas disease)	? [122]	? [122]
*Triatoma* sp.	*Trypanosoma* sp. (Chagas disease)	EST [123], [124] MOX [123], [125]	-
**Phiraptera (body lice)**			
*Pediculus* sp.	*Rickettsia* sp. (epidemic thyphus)	? [33]	? [33]
**Siphonaptera (fleas)**			
*Xenopsylla* sp.	*Pasturella* (bubonic plague)	? [126], [127]	? [126], [127]

Metabolic resistance: EST, enhanced esterase activity; GST, enhanced glutatione-S-transferase activity; MOX, enhanced p450 monoxygenase activity. Target site resistance: AChE, modification of the acetylcholinesterase; GAB, modification of the GABA receptors; SCH, modification of the sodium channels. ?, Insecticide resistance present but mechanism unknown or unconfirmed to the best of our knowledge.

**Figure 1:**
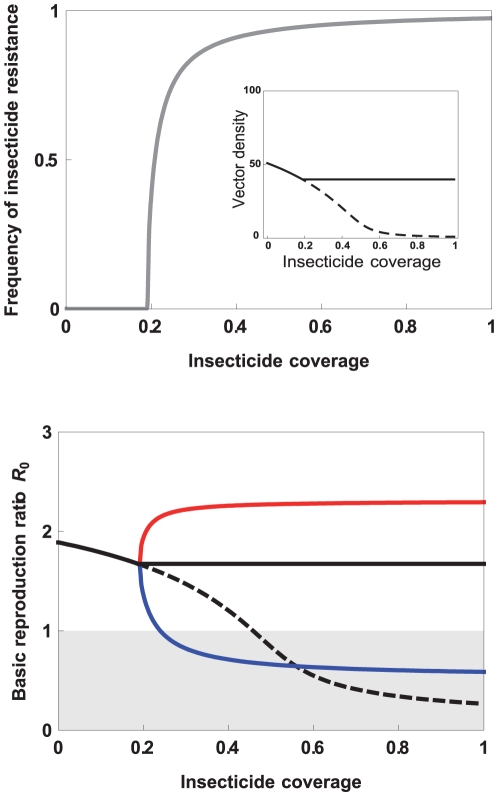
Effect of increasing insecticide coverage on (top) the frequency of insecticide resistance (IR, gray line), and, in the inset, the vector density with (full line) or without (dashed line) IR evolution; (bottom) the basic reproductive ratio of the infectious disease transmitted by the vector (see Box 1). (Bottom) We consider different scenarios: in the absence of IR evolution in the vector (dashed black line), and after IR evolution when the IR insects are equally good vectors as the susceptible ones (full black line), better (red line), or worse (blue line). The gray area delimits the area where the parasite goes to extinction (*R*_0_<1). See Appendix S1 for the details of the model and parameter values.

What is the impact of insecticide resistance on the transmission of vector-borne diseases? This can be best explored using a fundamental concept describing the transmissibility of infectious diseases: the parasite's basic reproductive number, or 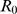 (see Box 1). This quantity plays a central role in epidemiology because it provides a synthetic index of transmission intensity and establishes threshold criteria for disease establishment or eradication. In particular, the prevalence of a disease is expected to increase in a naive population only when 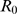 is greater than one. A major aim of insecticide spraying is to reduce the number of vectors (and thus *m* in Box 1). The emergence of insecticide resistance, however, counters this control method by increasing the number of mosquitoes that survive the insecticide treatment (Figure 1, top). This can result in substantial increases in vector numbers, possibly to pre-treatment densities (or nearly so, if there are costs associated with insecticide resistance [3]–[9]). Concerns about rebounding vector populations have been sufficient to motivate the search for novel insecticides [10], [11], the development of continent-wide resistance surveillance networks [12], [13], and work on resistance management strategies aimed at retarding or preventing the spread of resistance [14]–[18].

**Box 1. Basic Reproductive Number of Vector-Borne Diseases**

In the following, we distinguish the vector (e.g., mosquito in malaria) from the host (e.g., mammalian host in malaria). A general expression for *R*_0_ can be readily derived for simple vector-borne diseases [41], [117], [118]. We present the expression of *R*_0_ when the vector population is heterogeneous, consisting of both susceptible and resistant (prime symbol) individuals:
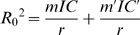
Where 1/*r* is the expected duration of the infection in the host (*r* is the rate of clearance of the infection in the host), *m* is the number of adult vectors per host, and *IC* is the *individual vectorial capacity* of the vector (modified from [119], [120]):
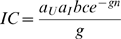
The other parameters are defined as follows:

*a_U_* and *a_I_*: number of (uninfected and infected bites) on the focal host, per vector and per day, which depends on *f_U_* and *f_I_*, the vector's feeding rates, and on *Q_U_* and *Q_I_*, the proportion of those bites on the focal host (i.e., human versus other mammals in human malaria) such that *a_U_* = *f_U_Q_U_* and *a_I_* = *f_I_Q_I_*.

*b*: probability that a host becomes infected from a bite of an infected vector (i.e., host susceptibility and vector infectiousness).

*c*: probability that a vector becomes infected from a bite on an infected host (i.e., vector susceptibility).

*g*: death rate of the vector. In other words, 1/*g* is the expected lifespan of a vector, and *e* ^−*g*^ is the probability a mosquito survives one day.

*n*: incubation time of the parasite in the vector (i.e., number of days required for the vector to become infectious after biting an infected host).

Insecticide resistance can have an effect on vector abundance (*m*′) but may also alter the vector's individual vectorial capacity (*IC*′) by modifying the vector's longevity (1/*g*′), competence (*b*′, *c*′, *n*′), and behaviour (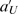 and 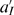).

One factor that has been largely overlooked is the potential effects of insecticide resistance on the ability of the vectors themselves to transmit disease (the *individual vectorial capacity*, Box 1): are insecticide-resistant insects better or worse vectors of diseases than susceptible ones? Far from being mere flying syringes, vectors provide a very specific environment in which the parasites differentiate, proliferate, and migrate to the correct tissues to ensure transmission to the next host. Recent work suggests that this environment is drastically modified when insects become resistant to insecticides [19], [20]. McCarroll et al. [21], [22] have shown that insecticide-resistant *Culex quinquefasciatus* mosquitoes are less likely to transmit the filaria parasite *Wuchereria bancrofti* than their insecticide-susceptible counterparts, and insecticide resistance in *Culex pipiens* seems to interact in a complex way with microsporidian and bacterial organisms [23]–[25]. Thus, increasing numbers of resistant insects need not lead to proportionate increases in disease transmission: it depends on whether those insects are more or less permissive transmitters than their susceptible ancestors. In this review, we survey a range of possibilities. We conclude that if the few data that exist extend to other combinations of vector species, insecticide resistance mechanisms, and parasites, it is currently not possible to evaluate the public health significance of insecticide resistance.

## Insecticide Resistance Mechanisms

Four classes of chemical insecticides are the mainstay of vector control programmes: organochlorines, organophosphates, carbamates, and pyrethroids [1]. More recently, two alternative insecticide types have been introduced, largely for the control of mosquito larvae: biopesticides (e.g., *Bacillus thuringiensis*, *Bacillus sphaericus*) and insect growth regulators, such as the juvenile hormone mimic, methoprene [1]. Cases of resistance to these alternative insecticides are still limited (but see [26]–[29]) and the underlying mechanisms are only beginning to be identified [30]–[32].

To date, four types of resistance mechanisms against the chemical insecticides mentioned above have been described: metabolic resistance, target site resistance, penetration resistance, and behavioural resistance. To illustrate our arguments, we focus on metabolic and target site resistance because they have been extensively investigated at both the genetic and molecular levels [33].

Metabolic resistance involves the sequestration, metabolism, and/or detoxification of the insecticide, largely through the overproduction of specific enzymes [34], [35]. Three main groups of enzymes have been identified (Table 1): carboxylesterases (efficient against organophosphate and carbamate insecticides), glutathione-S-transferases or GSTs (efficient against organophosphates, organochlorine, and pyrethroid insecticides) and cytochrome P450-dependent monoxygenases (efficient against most insecticide types, frequently in conjunction with other enzymes). The overproduction of these enzymes may be achieved via two non-exclusive mechanisms: gene amplification increasing the gene's copy number [35] and gene expression via modifications in the promoter region or mutations in *trans*-acting regulatory genes [35], [36]. In addition, in some mosquito species, carboxylesterase resistance to the insecticide malathion has been associated with a qualitative change in the enzyme (a few amino acid substitutions can increase the rate of hydrolysis of the enzyme [37]).

In contrast, target site resistance is achieved by point mutations that render the actual targets of an insecticide less sensitive to the active ingredient [33], [38]. Most insecticides developed to date are neurotoxic and aim for one of the following three targets: the acetylcholinesterase (whose role is the hydrolysis of the neurotransmitter acetylcholine), the γ-aminobutyric acid (GABA) receptors (chloride-ion neurotransmission channels in the insect's nervous system), or the sodium channels (responsible for raising the action potential in the neurons during the nerve impulses). The acetylcholinesterase is the target of organophosphorous and carbamate insecticides, the GABA receptors are the main targets of cyclodiene (organochlorine) insecticides, and the sodium channels are the targets of pyrethroid and organochlorine insecticides. Mutations in all three of these can confer resistance (Table 1).

## What Effects on Parasite Transmission?

The evolution of insecticide resistance entails a battery of correlated life history changes in the insect, which are widely thought to be the result of pleiotropic effects of the insecticide resistance genes themselves, or of genes closely linked with them as a result of hitchhiking. These life history changes are often, though not always [39], [40], associated with fitness costs [3]–[9], that is, reduced fitness in the absence of insecticides. The question with which we are concerned here is how these changes interfere with the infection, development, and transmission of the parasite. Aside from the effect on mosquito population density, insecticide resistance can impact all of the main mosquito-related parameters in 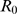 (given in Box 1). These include vector longevity, vector competence, and vector feeding behaviour. Below, we analyse each of them separately (see Table 2 for a summary).

**Table 2:** Potential effects of the different mechanisms of insecticide resistance (IR) on vector longevity, competence and behaviour, and expected effects on the parasite's *R*_0_.

Pleiotropic Effects of Insecticide Resistance	Mechanisms Concerned	Traits Affected	Effect on *R*_0_
**Vector longevity**			
IR trades off with resources needed to insure longevity	EST, GST, MOX	Decreased longevity (*1/g*)	Negative
IR increases oxidative stress	MOX, EST	Decreased longevity (*1/g*)	Negative
IR protects against oxidative stress	GST	Increased longevity (*1/g*)	Positive
**Vector competence**			
IR renders the vector toxic for the parasite	EST, MOX	Decreased probability of infection (*c*), decreased parasite growth and development (*b*)	Negative
IR blocks the immune response	GST	Increased probability of infection (*c*), increased parasite growth and development (*b*)	Positive
IR stimulates the immune response	EST	Decreased probability of infection (*c*), decreased parasite growth and development (*b*)	Negative
IR trades off with resources needed to insure immunity	EST, GST, MOX	Increased probability of infection (*c*), increased parasite growth and development (*b*)	Positive
IR trades off with resources needed for parasite development	EST, GST, MOX	Decreased parasite growth and development (*b*), increased parasite incubation time (*n*)	Negative
**Vector behaviour**			
IR alters the functioning of the nervous system	AChE, GABA, SCH	Hyperactive or sluggish vector: decreased or increased biting rate of the focal host (*a*)	Positive/Negative
IR trades off with resources needed for vector mobility	EST, GST, MOX	Sluggish vector: decreased biting rate of the focal host (*a*)	Negative
IR switches feeding preferences away from blood	EST, GST, MOX	Decreased biting rate of the focal host (*a*)	Negative

See Table 1 for acronyms.

### Insecticide Resistance and Vector Longevity

Vector longevity is an essential parameter in disease transmission, as it increases the potential for infective bites to hosts. As pointed out by MacDonald [41], the effect of longevity on disease transmission is particularly poignant for parasites like *Plasmodium* that need a minimum incubation period in the vector before being transmitted to a new host (Box 1). Yet, to our knowledge, there have been no thorough analyses on the effects of insecticide resistance on the longevity of *Anopheles* or indeed of any other vector of human disease, with one exception. In *C. pipiens*, insecticide resistance has been associated with a reduced longevity in the laboratory [24] and overwintering survival in the field [42], [43]. Similar effects of insecticide resistance on longevity have been obtained in other (non-vector) insect species [44]–[46]. Two main mechanisms may underlie this reduction in longevity: resource-based trade-offs and oxidative stress.

A well-known paradigm in evolutionary ecology is that diverting resources to one trait will, directly or indirectly, diminish the resources available for other traits [47]. This has been often put to the test using insect models, where it has been shown that, when resources are limited, an increased investment in certain fitness-associated traits such as fecundity is often coupled with a significant reduction in longevity [48], [49]. The deployment of insecticide resistance mechanisms, and in particular the overproduction of the detoxifying enzymes, likely requires substantial investment of resources. In the mosquito *C. pipiens*, for example, certain resistant genotypes can produce up to 50 times more esterases than their susceptible counterparts [50]. In other insects, these overproduced esterases can represent up to 3% of the total body proteins [51]. Lipids are likely victims of this large overinvestment in proteins, as they are an important source of the acetyl groups needed to synthesise the enzyme's constitutive amino acids [52]. Lipids are also the main fuel for insect survival [53], [54]. Unfortunately, so far as we are aware, no studies have quantified the level of lipids—or, indeed, any other energetic resource—in insecticide-resistant and -susceptible vectors.

Oxidative stress results from a mismatch between the production of damaging reactive oxygen species (ROS) and the production of protective antioxidants [55]. All organisms produce ROS as a result of the normal metabolic functioning of their cells [55]. The unwanted ROS produced in such reactions exert irreversible deleterious effects in the body [56] and have been widely proposed as a mechanism for ageing [55]–[57]. Blood feeding insects, in particular, face a considerable challenge from oxidative stress, because the digestion of haemoglobin results in a large production of ROS [58], [59]. In *Anopheles*, excess ROS production, though unrelated to insecticide resistance, has been recently shown to lead to a significant increase in mortality [60]. Two insecticide resistance mechanisms, in particular, may drastically alter ROS levels in insects, albeit in radically opposite ways: the p450 monoxygenases and the GSTs. The increased activity of p450 monoxygenases results in an excess production of harmful ROS because the stoichiometric demands of the enzymatic reaction are often not met [61]. This fact, previously known only from vertebrates [62], has been recently demonstrated in the house fly [63], and is thus likely to extend to other insect species. In contrast, GSTs have been shown to protect tissues against oxidative damage by increasing their solubility and aiding the excretion of free radicals [64]–[66]. A recent comparative study has found a clear association between GST expression and extended lifespan in fruit flies, nematodes, and mice [67]. Moreover, transgenic lines of *Caenorhabditis elegans* that produce 2.4 times more GSTs than controls show a 22% extension in their longevity [68]. The overexpression of GSTs in these transgenic worms is within the range found in insecticide-resistant vectors [69], [70]. Again, however, we are unaware of any studies addressing the longevity of vectors that are resistant to insecticides through the overproduction of GSTs.

### Insecticide Resistance and Vector Competence

Vector competence, the successful invasion and subsequent development of the parasite in the vector, depends on the plethora of physiological and immunological factors that determine the insect's internal environment for the parasite. Insecticide resistance could interfere with parasite development in at least two ways. First, the physiological modifications that accompany the deployment of insecticide resistance mechanisms may render the vector toxic to parasites. In one of the few studies to have explicitly investigated the connection between insecticide resistance and disease transmission, McCarroll and collaborators showed that the development of the filaria *W. bancrofti* larvae was arrested in insecticide-resistant *C. quinquefasciatus* mosquitoes [21], [22] (but see [71]). Exactly what rendered the insecticide-resistant mosquito toxic to the parasite is not known, but it was hypothesised that the overproduction of carboxylesterases (see Table 1) in these mosquitoes resulted in a change in the redox potential of the tissues hosting the parasite, which led to the death of the larvae. Pending confirmation of a correlation between carboxylesterase and ROS production, these results could extend to other parasites whose vector stages have been shown to be highly susceptible to oxidative stress (such as *Plasmodium* [72] and *Trypanosoma* [73]), as well as to other insecticide resistance mechanisms (such as the p450 monoxygenases and GSTs) with a proven link with oxidative stress (see above).

Second, insecticide resistance could affect vector immunity. The combined complexity of the mode of action and the multiple substrate specificities of the enzymes involved in metabolic insecticide resistance (see Box 1) is such that these enzymes may have pleiotropic effects on one of the many steps of the immune cascade, from the recognition of the parasite as foreign, to the transduction of the signal and the deployment of the killing mechanism [74]. Yet, aside from a microarray study that showed upregulation of certain immune-related genes in insecticide-resistant strains of *Anopheles gambiae* [20], there are no studies that explicitly investigate the potential effects of insecticide resistance on insect immunity. Here we suggest two as yet unexplored possibilities.

The first concerns the protective role of GSTs (Table 1) against the effects of ROS on the parasites. Inducible ROS are a key component of the immune defence of *Anopheles* mosquitoes against *Plasmodium* [60], [75]. By neutralising the oxidative response of the mosquito to the parasite, overproduced GSTs could potentially increase the susceptibility of mosquitoes to the parasite.

The second concerns carboxylesterases (Table 1). Due to the overlapping substrate specificities existing between these enzymes and the serine proteases implicated in the melanization cascade, it has been suggested that carboxylesterases could have a positive effect on the formation of a melanin capsule around the parasite [76]. Two decades ago, an interesting association was found between an allele in an esterase locus and resistance by encapsulation in the G3 strain of *A. gambiae* infected with the B strain of *Plasmodium cynomolgi* [77]. The product of this gene was found to be a carboxylesterase with considerable sequence similarity to the carboxylesterase overproduced by insecticide-resistant *Culex* mosquitoes [78]. Subsequent (unpublished) studies, however, did not find any pattern of association between the carboxylesterase phenotype and *Plasmodium* susceptibility [79], but, to our knowledge, the question has not been investigated any further. More recently, carboxylesterases have been shown to be inducibly produced after bacterial [80] and viral [81] infections, suggesting that they may play a direct role in the invertebrate immune system. Thus, it is possible that upregulation of carboxylesterases as an adaptation against insecticides could, as an incidental side effect, make mosquitoes more resistant to pathogens.

Immunocompetence could also be affected through resource-based trade-offs. There is plenty of evidence that there are significant resource costs involved in the deployment and maintenance of the insect immune system [82]. Proteins seem to be the limiting resource for the encapsulation and antimicrobial responses in caterpillars [82], [83], and lipid metabolism has been shown to be implicated in the immune response of *Aedes aegypti* mosquitoes to a *Plasmodium* and a bacterial infection [84]. The production of large amounts of detoxifying enzymes, such as esterases or GSTs, is likely to deplete the resource pool, limiting the vector's ability to mount an immune response, therefore favouring the development of the parasite. It is worth noting, however, that resource limitation could also have the opposite effect if redirection of resources to insecticide resistance puts those resources beyond the reach of parasites: it could limit the development of parasites that depend on the host's energetic reserves to fulfil their own metabolic needs [85]. In vitro studies have, for example, shown that the mosquito gut stages of *Plasmodium* are greedy consumers of amino acids [86], lipids [87], and glucose [88]. There is also evidence that parasite production is positively correlated with resource availability in several invertebrates [89]–[92]. In these systems, the redirection of resources towards insecticide resistance is likely to impair the ability of the parasites to develop inside the vectors.

### Insecticide Resistance and Vector Behaviour

Vector behaviour, particularly host choice, and biting rate have key effects on parasite transmission (Box 1). Mosquitoes with transmissible stages of *Plasmodium* persist at biting for longer than uninfected mosquitoes or mosquitoes infected with non-infectious stages [93]. Similar results have been obtained with *Leishmania*-infected sandflies [94]. In addition, recent work shows that uninfected mosquitoes are preferentially attracted to humans infected with transmissible gametocytes [95]. Because of its direct effect on the vector's neural system, target site resistance in particular has the potential for modifying the biting behaviour of uninfected and infected vectors alike.

Target-site resistance mutates key components of the vector's neural network, drastically modifying their performance and, thus, potentially also their response to external stimuli. In *C. pipiens*, for example, the single point mutation that renders the acetylcholinesterase insensitive to insecticides reduces the activity of the enzyme by up to 60% [96], which is likely to result in an excess of acetylcholine in the synapses and in a hyperactivity of the nervous system [6]. The most compelling examples of the effect of target site resistance on insect behaviour have not been carried out in vectors of diseases but on aphids and flies. In these insects, the *kdr* mutations alter the normal functioning of the sodium channels, causing a reduction in the excitability of the nervous system [97], [98]. Consequently, *kdr*-resistant aphids are less responsive to the presence of pheromone released by conspecifics [98], [99], increasing their vulnerability to parasitoid attack [100]. Furthemore, *kdr*-resistant flies are also less responsive to changes in temperature gradient than their insecticide-susceptible counterparts [98]. In mosquitoes, sodium channels are implicated in the transduction of the olfactory signal from the olfactory receptors to the central nervous system [101]. Target-site modifications, such as the *kdr* mutation, may render mosquitoes less responsive to the olfactory cues, such as lactic acid or ammonia [102], [103], that allow them to locate their hosts, thus reducing their efficiency as vectors. Rowland [104], [105] found that target-site resistance to organochlorine insecticides rendered *A. gambiae* and *Anopheles stephensi* mosquitoes less responsive to oviposition and predation-risk stimuli, but the effects on blood feeding behaviour have, to our knowledge, never been investigated.

Perhaps less intuitively, however, the behavioural side effects of insecticide resistance also extend to metabolic resistance. Foster et al. [98] showed that, in aphids, insecticide resistance through increased carboxylesterase titres were associated with a reduced ability to move away from senescing leaves. Berticat et al. [4] found that adults of *C. pipiens* that are resistant to insecticides through the overproduction of carboxylesterases suffered higher predation rates than susceptible ones, probably due to a decreased locomotive performance. This seemingly decreased mobility of insecticide-resistant insects is likely to be the result of resource depletion associated with the overproduction of carboxylesterases [98]. When applied to a blood-feeding vector, reduced motility may translate into reduced host-seeking efficiency and biting rates, although this has never been tested. A decrease in the energetic reserves may also switch the feeding preference of vectors away from hosts. In *Ae. aegypti* and *Culex nigripalpus* mosquitoes, resource deprivation, which is directly correlated with low energetic reserves, renders mosquitoes more responsive to sugar-rich odours like honey and less responsive to host odours [106].

## Discussion

Whether a particular insect is a good vector, an occasional vector, or whether it presents an infection barrier for the parasite depends on a plethora of physiological, immunological, and behavioural variables. In this review, we have argued that any of these factors may potentially be altered by the evolution of insecticide resistance, with potentially drastic consequences for the epidemiology of disease (Figure 1). If insecticide resistance decreases the individual vectorial capacity of the vector (blue line in Figure 1), the transmission of the disease can decrease below the level attained in the absence of insecticide resistance evolution. In this case, insecticide resistance evolution may thus decrease the level of insecticide coverage needed to drive the parasite to extinction. An increase in the individual vectorial capacity (red line in Figure 1), on the other hand, may lead to a dramatic increase in the transmission of the disease and even to a higher prevalence than in the absence of insecticides. Moreover, even when local eradication does not occur (perhaps because initial 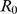 is very high), the extent to which the very impressive disease control often achieved by insecticides is eroded as resistance spreads will depend not only on how vector densities recover but also on the vectorial capacity of individual vectors, which, as we have argued, can be dramatically altered by resistance. As is clear from our discussion above, surprisingly little work directly addresses this important issue. Below, we summarise what we consider to be the three main questions to be answered, and we outline some predictions that arise from the mode of action of the different insecticide resistance mechanisms.

The first question is whether insecticide-resistant vectors have a different lifespan than their susceptible counterparts. We expect that, in most cases, the effect of insecticide resistance will be to reduce vector longevity. This has been already shown in insects of agricultural interest as well as in *Culex* mosquitoes [24], [42]–[46], but it needs to be tested in the other vectors of diseases, most particularly those that transmit parasites with long incubation periods (e.g., the mosquitoes *Anopheles* and *Aedes*, and the kissing bugs *Rhodnius* and *Triatoma*) (Table 1). We further expect this longevity reduction to be especially drastic in insects with metabolic insecticide resistance as a result of resource-based trade-offs and/or increased oxidative stress. The one exception to this rule may be vectors overexpressing the GST, which has been shown to increase lifespan in organisms as diverse as *Drosophila* and nematodes [67], [68]. The longevity reduction in insecticide-resistant insects may, however, be offset by the parasite's influence on longevity. In *C. pipiens* mosquitoes infected with the microsporidia *Vavraia culicis*, the decrease in longevity associated with insecticide resistance is much larger for uninfected than for infected mosquitoes [24]. Indeed, parasites often have an effect on the longevity of their vectors, both positive and negative [107]. Thus, whenever possible, the potential interaction between insecticide resistance and parasite-mediated effects on the vector's lifespan needs to be investigated, ideally, using natural vector–parasite combinations [107], [108].

The second question is whether insecticide resistance alters the probability an insect becomes infected and/or the subsequent intensity of infection and production of transmission stages (or vector competence). McCarroll and co-workers [21], [22] have shown that insecticide-resistant mosquitoes have lower burdens of filaria parasites, possibly due to an increase in oxidative stress. Vontas et al. [109] failed to show differences in parasite burden between insecticide-resistant and -susceptible *An. stephensi* mosquitoes infected with *Plasmodium yoellii,* although the different geographic origin of the resistant and susceptible strains and the unnatural combination of an Asian vector with an African rodent parasite make these results difficult to interpret (see below). We expect the effects on parasite burden to be more drastic in vectors with metabolic resistance, as the production of large amounts of detoxification enzymes will likely render the physiological environment of the vector less than ideal for parasite development. Unfortunately, a mere reduction in parasite burden in insecticide-resistant insects is unlikely to have a drastic effect on disease transmission because, in most cases, a few parasites suffice to initiate a new infection in the host. As few as ten *Plasmodium* parasites are sufficient to establish a malaria infection [110]. One way in which parasite burden may influence transmission, however, is if it correlates with vector survival. There indeed is evidence, again from *Plasmodium*, that more heavily infected mosquitoes die faster [108], [111].

The third question is whether insecticide resistance modifies the biting rate or host choice of the uninfected and/or infected vector. We expect this effect to appear particularly in vectors that are resistant through modifications in the neural targets of the insecticide, because of the obvious connections between behaviour and the nervous system. Depending on the underlying mechanism, these modifications may result in either a hyperactive or a sluggish nervous system, but how this translates into feeding and host-choice behaviour remains to be investigated. Finally, of particular interest is whether insecticide resistance may be able to alter the parasite-mediated manipulation of vector feeding behaviour, even though, in most cases, this manipulation takes place through a physical interference with blood ingestion [107], without the involvement of the nervous system.

As illustrated above, the physiological mechanisms underlying insecticide resistance yield clear predictions as to how insecticide resistance may affect the different components of the parasite's 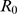 (vector longevity, competence, and behaviour, Table 2). However, the same insecticide resistance mechanism may have opposite effects on each of these components by, for instance, increasing the vector's lifespan but interfering with the parasite's development (see GST, Table 2). It is therefore difficult to predict the overall effect of insecticide resistance on a parasite's 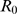. In addition, our predictions in Table 2 probably do not encompass all possible effects of insecticide resistance on disease transmission. The enzymes involved in the detoxification of insecticides belong to particularly complex families of enzymes whose substrate specificities and biological functions are not yet fully known [112]. Similarly, target-site mutations seem to have pleiotropic effects that go beyond the nervous system [113]. The problem of prediction gains additional intricacy from the fact that many insect vectors are now resistant to multiple insecticide types through a combination of different metabolic and target-site modification mechanisms [114], [115]. These different insecticide resistance mechanisms have been shown to interact with each other [6], but what consequences these interactions may have for parasite transmission will have to be resolved on a case-by-case basis.

Studies investigating the vectorial capacity of insecticide-resistant and -susceptible vectors are, in our view, urgently needed, but we note three experimental challenges that need to be overcome in order to reach strong conclusions. The first is that single comparisons of allopatric-resistant and -susceptible vector strains [19], [20], [109], [116] cannot disentangle the effects of insecticide resistance genes from other differences that inevitably arise during divergent evolutionary history. Much stronger inferences can be made if sympatric-resistant and -susceptible mosquitoes are compared, but in areas with a long and complex history of insecticide use, fully susceptible individuals can be very hard to find. If obtaining matched sympatric lines is not feasible, many unmatched resistant and sensitive lines are required. Another way forward is the comparison of laboratory-selected lines. But this raises a second experimental difficulty: the conclusions from laboratory-selected, insecticide-resistant strains may not be directly applicable to the conditions in the field. Curtis [21], [71] and McCarroll et al. [21] pointed out that McCarroll's [22] results with *Culex* and *Wuchereria* may have been the result of unnaturally high esterase levels in the laboratory-selected strains of the mosquito. In addition, while selecting for insecticide resistance in the laboratory, one may inadvertently select for other traits, such as developmental time, body size, immunocompetence, or longevity, which may have consequences for parasite transmission. The final experimental issue is that, whenever possible, studies should be carried out on natural vector–parasite combinations. Lessons from *Plasmodium* studies have taught us that results obtained using laboratory models, most notably concerning mosquito longevity [108] and immunity [117], are not necessarily applicable to natural vector–parasite combinations. We agree that overcoming all three of these pitfalls is not easy, but the logistic difficulties do not mean the problems can be ignored.

Thus far, we have concentrated our discussion on the short-term effects of insecticide resistance on parasite transmission through its impact on the parasite's 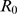 (epidemiological time scale). However, the interaction between the parasite and the insecticide-resistant vector can also have long-term (evolutionary time scale) consequences. Insecticide resistance could exert a selective pressure for the evolution of the parasites by selecting for parasites with, for example, shorter incubation times (to compensate for the reduction in longevity), or faster multiplication rates (to compensate for higher parasite mortality). Conversely, if parasite burden is reduced in insecticide-resistant vectors, as McCarroll et al. [22] showed, this could facilitate the spread of insecticide resistance in vector populations submitted to a significant parasite pressure. Exploring these two evolutionary consequences is beyond the scope of this paper, but the interaction between insecticide resistance and parasitism clearly deserves further investigation.

Insecticide resistance is generally thought to undermine the control of vector-transmitted diseases. Consequently, there are ongoing efforts to develop resistance-breaking compounds [10], [11] and evolution-proof insecticidal strategies [14], [15], as well as improved resistance surveillance in the field [12], [13]. We suggest that another research problem be added to this agenda: the disease transmission capacity of resistant insects. In some instances, insecticide resistance may impair the ability of the vector to transmit diseases. If this effect is sufficiently large, the impact of insecticide resistance on disease management may not be as detrimental as previously thought. If so, current paradigms might be leading to a misallocation of research and control resources. We contend that there are surprisingly few well-documented cases of disease outbreaks in response to the evolution of insecticide resistance (in marked contrast to the well-documented public health problems caused by the evolution of drug resistance). Alternatively, insecticide resistance could improve the individual vectorial capacity of insects, further emphasising the urgent need for novel insecticides and resistance management strategies. Either way—and there may be no simple generality—the consequence of the evolution of insecticide resistance for disease ecology deserves additional attention.

## Supporting Information

Appendix S1 The Model Presented in Figure 1
(0.09 MB DOC)
