# Supplementary material for: Insecticide Control of Vector-Borne Diseases: When Is Insecticide Resistance a Problem?
Source: PLoS Pathog. 2010 Aug 5;6(8):e1001000. doi: 10.1371/journal.ppat.1001000 (PMC2916878; doi:10.1371/journal.ppat.1001000)
Supplement: Appendix S1 — The Model Presented in Figure 1 (0.09 MB DOC) [file ppat.1001000.s001.doc]

Appendix 1│ **The model presented in figure 1.**

The evolutionary epidemiology of insecticide resistance is modeled under the following assumptions. In the absence of insecticides, we assume a logistic growth of the vector population. The susceptible vectors ($V$ denotes the density of susceptible vectors) reproduce and die at rates $\lambda$ and $d$, respectively, and $K$ is the carrying capacity of the vector population. We further assume an immigration rate $m$ of susceptible vectors in the population. The insecticide is assumed to reduce the fecundity of susceptible vectors (larvicide) and $i$ measures the coverage of insecticides ($i$ varies between 0 and 1). In contrast, resistant vectors ($R$ denotes the density of resistant vectors) do not suffer from the effect of insecticides but pay a cost of resistance $c$on adult survival. This yields the following equations:

$$\begin{matrix} \frac{dV}{dt}=\mu+\lambda\left(1-i\right)V\left(1-\frac{V+R}{K}\right)-dV \\ \frac{dR}{dt}=\lambda V\left(1-\frac{V+R}{K}\right)-d(1+c)V \end{matrix}$$

In figure 1 we used the following parameter values: $\lambda=2$, $d=1$, $K=100$, $\mu=1$, $c=0.2$. Figure 1a presents the effect of insecticide coverage on (1) migration-selection equilibrium of the frequency of resistance, and (2) the equilibrium vector density (i.e. $V+R$) with or without resistance evolution. Figure 1b presents the effect of insecticide coverage on the basic reproduction ratio $R_{0}$ (see box1), with $m=\frac{V}{H}$ and $m'=\frac{R}{H}$ , where $H$ is the density of human hosts. In this figure we further assumed that $\frac{IC}{rH}=0.07$ and . The parameter $\varepsilon$ thus measures the effect of insecticide resistance on the individual vectorial capacity (i.e. the “quality” of the vector) relative to susceptible vectors. When $\varepsilon>0$ ($\varepsilon<0$) IR individuals are better (worse) vectors. In figure 1b we used three values of $\varepsilon$: $\varepsilon=0$ (black curve), $\varepsilon=0.9$ (red curve), $\varepsilon=-0.9$ (blue curve). The actual parameter values chosen in these figures do not matter much here, as we only want to illustrate the effect of a variation of the quality of IR vectors on the epidemiology of the disease.
